# Low Apgar score and asphyxia complications at birth and risk of longer-term cardiovascular disease: a nationwide population-based study of term infants

**DOI:** 10.1016/j.lanepe.2022.100532

**Published:** 2022-11-03

**Authors:** Neda Razaz, Mikael Norman, Tobias Alfvén, Sven Cnattingius

**Affiliations:** a Clinical Epidemiology Division, Department of Medicine Solna, Karolinska Institutet, Stockholm, Sweden; b Department of Clinical Science, Intervention, and Technology, Division of Pediatrics, Karolinska Institutet, and Department of Neonatal Medicine, Karolinska University Hospital, Stockholm, Sweden; c Department of Global Public Health, Karolinska Institutet, Stockholm, Sweden; d Sachs' Children and Youth Hospital, South General Hospital, Stockholm, Sweden

**Keywords:** Asphyxia-related complication, Low Apgar Score, Neonatal seizures, Cardiovascular diseases, Cerebrovascular diseases

## Abstract

**Background:**

Most follow-up studies have focused on the long-term consequences of asphyxia at birth on the brain. The aim of this study was to investigate associations between low Apgar score and asphyxia-related complications and subsequent risks of cardiovascular diseases (CVD) in childhood and early adulthood.

**Methods:**

This population-based cohort study included 2,826,424 non-malformed singleton births, born at term (≥37 weeks’ gestation) between 1988 and 2018 in Sweden. Primary exposure was a composite of asphyxia-related complications, defined as a) Apgar score 0–3 at 1-min; or b) Apgar score 0–3 at 5-min; or c) neonatal seizures (including hypoxic ischemic encephalopathy). Using Cox regression, we estimated the risk of CVD after 1 year of age, defined as stroke, coronary heart disease, heart failure, and atrial fibrillation.

**Results:**

Overall, there were 4165 cases with cardiovascular diseases. Individuals with asphyxia-related complications had adjusted hazard ratios (95% confidence intervals) of 1.90 (1.54 to 2.34) for cardiovascular disease, 2.29 (1.74 to 3.03) for stroke, 2.17 (1.37 to 3.42) for heart failure, and 1.38 (0.87 to 2.17) for atrial fibrillation. Hazard ratios for CVD were elevated among individuals with Apgar score 0–3 at 1 and 5 min, and those with neonatal seizures. Compared with unexposed individuals, neonatal seizures were associated with 5 times higher rates of stroke and heart failure, respectively.

**Interpretation:**

Asphyxia-related complications and its neonatal complications, especially low Apgar score and neonatal seizures, are associated with increased risks of CVD in childhood and early adulthood, although the absolute risk of CVD is low in young age.

**Funding:**

10.13039/501100004359Swedish Research Council and the 10.13039/501100003793Swedish Heart-Lung Foundation

**Research in context**

**Evidence before the study**

We searched PubMed for articles assessing risk of cardiovascular diseases in relation to low Apgar score and asphyxia-related complications at birth up to March 1, 2022, using the search terms “asphyxia”, “birth asphyxia”, “Apgar score”, “cardiovascular disorders”, “childhood cardiovascular diseases” and “risk”. We found no observational study examining the relationship between asphyxia-related complications and risk of cardiovascular disorders. Previous follow-up studies have focused on the long-term neurodevelopmental consequences of asphyxia, but we found no observational study examining the relationship between asphyxia-related complications and risk of cardiovascular disorders.

**Added value of the study**

In this population-based study, non-malformed term (≥37 weeks) infants experiencing asphyxia-related complications, especially infants with low Apgar scores (0–3) at 5 min and those with neonatal seizures, have increased risks of cardiovascular diseases (CVD) in childhood and early adulthood. In particular, increased risks were observed for stroke and heart failure.

**Implications of all available evidence**

Although the absolute risk of CVD is low in young age, the present findings provide support that asphyxia-related complications and its neonatal complications, especially low Apgar score and neonatal seizures, are associated with cardiovascular morbidity later in life. Our findings need to be replicated, and possible underlying mechanisms, linking asphyxia-related complications to CVD, need to be identified.

## Introduction

Cardiovascular diseases are the leading causes of disability and death in most countries.^1^ Besides genetic predisposition and adult life-style, low and high birth weight,^2^ preterm birth,^3^^,^^4^ maternal smoking in pregnancy,^5^ gestational diabetes,^6^ and obesity^7^ have been identified as perinatal risk factors influencing the subsequent risks of cardiovascular development in early adulthood. However, while risk factors for cardiovascular diseases so far reflect chronic exposures and adaptations, there is a lack of evidence whether acute perinatal complications also increase the risk of cardiovascular diseases in adulthood.

Asphyxia is characterized by tissue hypoxia and energy-failure that arises due to ischemia, globally occurs in about 4 million babies every year, approximately 1 per 1000 live births.^8^^,^^9^ Our recent study demonstrated that there is a dose–response relationship between maternal obesity severity and risk of cardiovascular diseases in childhood and early adulthood.^7^ We also found that 22% of this relationship was mediated by neonatal complications after birth asphyxia.^7^ Asphyxia at birth commonly leads to neonatal hypoxic-ischemic encephalopathy (HIE) which in severe forms is characterized by initial cell death, followed by remodeling, scarring and repair.^10^ The reported incidence of HIE, ranges from about 1.7 per 1000 live births in developing countries and as high as 26 per 1000 live births in developing nations.^11^

Much of the research has been concentrated on long-term neurodevelopmental impairments after asphyxia, such as cerebral palsy,^12^ epilepsy and cognitive problems - which affects one third of infants with moderate (stage 2) HIE and almost 100% in infants with severe (stage 3) HIE.^13^ Between 25 and 60% of infants suffering from birth asphyxia exhibit clinical evidence of cardiac complications in the neonatal period.^14^ Although hypoxic-ischemic injury also affects the cardiovascular system, we are unaware of any study investigating associations between asphyxia-related conditions at birth and risk of long-term cardiovascular disease. We therefore performed a nation-wide cohort study, including more than 2.8 million term (≥37 weeks of gestation) infants born in Sweden, to investigate the associations between low Apgar score and asphyxia-related complications and risks of cardiovascular diseases in children and young adults.

## Methods

This population-based cohort study included all live singleton births, born at ≥37 completed gestational weeks, who were recorded in the Swedish Medical Birth Register from January 1, 1988 to December 31, 2018. Using the person-unique national registration numbers of mothers and children,^15^ the Medical Birth Register was linked with the National Patient-, Cause of Death-, Total Population-, Education-, and Multi-generation Registers. The Medical Birth Register includes prospectively recorded information from prenatal, obstetric, and neonatal care on more than 98% of all births in Sweden.^16^ The National Patient Register includes nation-wide diagnostic information on hospital admissions since 1987 and hospital out-patient care from 2001.^17^^,^^18^ The Cause of Death Register contains information of all deaths in Sweden.^19^ The Total Population Register includes information on country of birth.^20^ The Education Register records the highest level of education and is updated yearly.^21^ The Swedish Multi-generation Register includes information on the personal registration numbers of first degree relatives, and was used to obtain information of the fathers’ personal identification numbers. Diagnoses are coded according to the International Classification of Diseases (ICD). Sweden used the ninth revision (ICD-9) from 1987 through 1996, and the tenth revision (ICD-10) has been used since 1997. The study was approved by the Ethics Review Authority in Sweden (No. 2020-01545).

### Study population

From 1988 through 2018, the Medical Birth Register recorded information for 3,181,285 live singleton births. We excluded 4108 births where either the mothers or the children had invalid national registration numbers, 151,845 preterm born singletons, 9593 who died before their first birthday, 8745 children who emigrated before one year of age (start of follow-up), 125 with missing information on child's sex or maternal age, 2463 who had an ICD code for the outcome during the first year of life, and 180 445 infants with congenital malformation diagnoses during the study follow-up time (see Table S1 for specific ICD codes). Among those who died within the first year of life 3492 (36.4%) had asphyxia-related complications and 473 (4.9%) had CVD. Rates of CVD events in children with and without congenital malformations are provided in Table S2. After these exclusions, 2,826,424 live non-malformed term singleton births were included in the study.

### Exposure

Our primary exposure was a composite of asphyxia-related complications diagnosed in the first 27 completed days of life (0–27 days), captured from the Medical Birth Register and the National Patient Register. Asphyxia-related complications was defined as a) Apgar score 0–3 at 1 min or; b) Apgar score 0–3 at 5 min or; c) seizures (including hypoxic ischemic encephalopathy)^22^ in the neonatal period (see Table S1 for specific ICD codes). A composite exposure of asphyxia-related complications was the presence (vs absence) of any one of these conditions. We further examined each condition separately. The Swedish neonatal intensive care units practice neurophysiological methods (amplitude-integrated EEG monitoring or conventional electroencephalogram) to diagnose neonatal seizures or other signs of encephalopathy as recommended by national guidelines for neonatal seizure management.^23^, ^24^, ^25^ Table S3, displays the overlap between the exposures.

### Outcomes

The primary outcome was the first cardiovascular disease (CVD) event recorded after 1st year of life. Outcome information was obtained by using ICD codes in the National Patient Register or the Cause of Death Register from 1989 through 2020, including primary or secondary diagnoses. The primary outcome was cardiovascular disorders of any type (coronary heart disease [ICD-9: 410–414, ICD-10: I20–I25]; heart failure [ICD-9: 428, ICD-10: I50]; stroke [ICD-9: 430–436, ICD-10: I60–I65]; and atrial fibrillation [ICD-9: 427D, ICD-10: I48. To avoid measuring CVD as a neonatal complication (such as neonatal stroke), all individuals with recorded ICD codes for CVD before 1 year of age were excluded from the study. The outcomes were not mutually exclusive. In addition, associations between asphyxia-related complications and subgroups of cardiovascular diseases were considered.

### Covariates

All women in Sweden are offered ultrasound dating of pregnancy no later than early in the second trimester, and 95% of the women accept this offer.^26^ Gestational age was estimated in completed weeks using the following hierarchy: the date of early second trimester ultrasound (87.7%), the date of the last menstrual period (7.4%), or a postnatal assessment (4.9%). Maternal characteristics included age at delivery, country of birth, education, cohabitation with a partner, parity, height, body mass index (BMI, kg/m^2^), and smoking during pregnancy. Maternal BMI in early pregnancy was calculated from self-reported height and weight measured at the first antenatal visit, which occurs within the first 14 weeks of gestation (ie, first trimester). Mothers who reported daily smoking at the first prenatal visit and/or at 30–32 gestational weeks were classified as smokers. Birth weight-for-gestational age was estimated using the sex-specific Swedish reference curve for normal fetal growth.^27^ Mode of delivery was recorded in standardized checkboxes as non-instrumental or instrumental vaginal delivery, emergency or elective cesarean section. Maternal and paternal cardiovascular diseases were defined using the same definitions as for children.

### Statistical analysis

Results were presented as incidence rates per 10,000 person years with 95% confidence intervals (CIs) for CVD according to asphyxia-related complications. Cumulative hazard curves were used to compare rates of CVD over time according to asphyxia-related complications as a composite and each component separately. The primary and secondary outcomes of time to first CVD event were analyzed with Cox proportional hazard regression models with age as the time scale, and are presented as crude and adjusted hazard ratios (HR). The time to first CVD event was calculated from one year of age until first diagnoses of CVD, death, loss to follow-up (e.g. emigration), or end of study (December 31, 2020), whichever came first. All HRs are presented with 95% CI.

First, rates of CVD were compared between categories of maternal and neonatal characteristics. Next, rates of CVD within each asphyxia-related complication were estimated. Adjusted HRs were obtained from multivariable Cox models with covariates, including maternal age, parity, mother's country of origin, education level, cohabitation with partner, height, smoking, child's sex, and year of delivery. In addition, associations of asphyxia-related complications with CVD subtypes were examined. As clustered robust standard errors (to account for clustering of siblings) only changed confidence intervals in the third decimals, confidence intervals are reported with normal standard errors without adjustment for clustering. In sensitivity analyses, additional adjustments were made for birth weight. As maternal BMI was missing in 542,408 (19.2%) women, adjustment for maternal BMI was only carried out in sensitivity analyses. We also tested the cross-product interaction terms between asphyxia-related complication and child's sex with the use of a Wald χ2 test. All analyses were carried out with the use of Statistical Analysis Software version 9.4 (SAS Institute, Cary, NC).

### Role of the funding source

The funders had no role in the study design, data collection, analysis, interpretation, writing the report, or the decision to submit the manuscript for publication. The corresponding author had full access to the data and had final responsibility for data integrity and data analysis, and the decision to submit for publication.

## Results

With a total of 44,998,488 million accumulated person-years, the median follow-up time was 15.9 (interquartile range [IQR], 7–24) years for exposed and 15.4 (IQR 7–25) years for unexposed individuals. The median age at diagnoses of CVD was 20.3 years. During the follow-up time, we recorded 4165 CVD events; 471 (11.3%) were diagnosed with CVD between 1 and 4 years, 859 (20.7%) between 5 and 16 years, 888 (21.3%) between 17 and 20 and 1937 (46.5%) and between 21 and 32 years (Table S4).

Low maternal education, maternal obesity (BMI ≥30 kg/m^2^), maternal smoking, vaginal instrumental delivery, elective and emergency cesarean delivery, male sex, low birth weight for-gestational-age (<3rd percentile), or high birth weight for-gestational-age (97th percentile) were associated with increased rates of CVD (Table 1, Table S5).

**Table 1:** Incidence rates of cardiovascular disease according to maternal characteristics, diseases, pregnancy complications, and neonatal characteristics.

Characteristics	Cardiovascular disease			
	No. of children	Child years of follow-up	No. of cases	Rate/10,000 child-years
**Total**	2,826,424	44,998,488	4165	0.93
**Maternal age (years)**				
≤19	50,742	945,717	122	1.29
20-24	435,878	7,905,304	916	1.16
25-29	940,269	15,700,955	1477	0.94
30-34	902,787	13,562,978	1129	0.83
≥35	496,748	6,883,535	521	0.76
**Country of birth**				
Sweden	2,265,164	37,843,155	3578	0.95
Other Nordic	64,550	1,159,506	134	1.16
Non-Nordic	487,574	5,807,485	433	0.75
Data missing	9136	188,343	20	1.06
**Education (years)**				
≤9	246,702	3,925,771	469	1.19
10-11	520,233	10,955,467	1260	1.15
12	654,376	9,595,169	829	0.86
13-14	425,865	7,011,984	649	0.93
≥15	962,931	13,413,417	949	0.71
Data missing	16,317	96,680	9	0.93
**Mother cohabits with partner**				
Yes	2,514,982	39,957,151	3636	0.91
No	151,230	2,240,591	260	1.16
Data missing	160,212	2,800,746	269	0.96
**Parity**				
1	1,198,376	18,808,403	1757	0.93
2	1,041,805	16,494,123	1469	0.89
3	408,402	6,764,827	650	0.96
≥4	177,841	2,931,136	289	0.99
**Maternal height (cm)**				
≤159	359,187	5,434,147	478	0.88
160-164	702,170	11,082,436	947	0.85
165-169	807,985	12,870,507	1182	0.92
≥170	864,247	13,503,061	1277	0.95
Data missing	92,835	2,108,336	281	1.33
**Maternal BMI**				
<18.5	64,028	1,044,039	106	1.02
18.5–24.9	1,430,040	21,368,154	1748	0.82
25–29.9	550,104	7,411,448	601	0.81
30–34.9	172,766	2,113,357	165	0.78
≥35	67,078	752,534	71	0.94
Missing	542,408	12,308,957	1474	1.20
**Smoking**				
No	2,369,137	35,821,350	3004	0.84
Yes	337,738	7,014,114	939	1.34
Data missing	119,549	2,163,024	222	1.03
**Diabetic disease**				
No	2,788,210	44,468,162	4116	0.93
Gestational diabetes	28,053	379,656	35	0.92
Pregestational diabetes	10,161	150,670	14	0.93
**Hypertensive disease**				
No	2,748,541	43,781,740	4046	0.92
Pregestational hypertension	16,026	240,165	19	0.79
Preeclampsia	61,857	976,583	100	1.02
**Maternal cardiovascular disorder**				
No	2,818,642	44,917,964	4161	0.93
Yes	7782	80,524	<5^a^	0.50
**Paternal cardiovascular disorder**				
No	2,809,921	44,819,569	4150	0.93
Yes	16,503	178,920	15	0.84
**Year of delivery**				
1988–1994	714,929	19,541,321	2701	1.38
1995–1999	390,366	8,554,402	697	0.81
2000–2004	395,486	6,726,206	369	0.55
2005–2009	445,271	5,431,249	207	0.38
2010–2014	480,899	3,553,335	144	0.41
2015–2018	399,473	1,191,975	47	0.39
**Mode of delivery**				
Vaginal non-instrumental	2,015,675	29,563,825	2231	0.75
Vaginal instrumental	176,451	2,575,146	224	0.87
Elective cesarean section	167,806	2,148,110	174	0.81
Emergency cesarean section	167,989	2,223,178	180	0.81
Data missing	298,503	8,488,230	1356	1.60
**Newborn's sex**				
Male	1,437,462	22,960,545	2672	1.16
Female	1,388,962	22,037,943	1493	0.68
**Birth weight for gestational age (percentiles)**				
<3	36,862	626,964	87	1.39
3 to <10	132,672	2,122,394	234	1.10
10 to 90	2,313,611	36,727,078	3302	0.90
>90 to 97	239,976	3,867,116	364	0.94
>97	98,068	1,563,940	164	1.05
Data missing	5235	90,996	14	1.54

Term singleton non-malformed live births in Sweden, 1988–2018.

a Exact count <5 is suppressed for confidentiality reasons.

The overall absolute rate of CVD was 0.93 per 10 000 person-years. In individuals exposed to asphyxia-related complications, absolute rate for CVD was 1.80 per 10,000 person-years. In individuals with Apgar score 0–3 at 1 and 5 min, and neonatal seizures, absolute rates were 1.34, 2.13, and 4.27 per 10,000 person-years, respectively (Table 2).

**Table 2:** Hazard ratios (HRs) and 95% confidence intervals (CIs) for cardiovascular disease among individuals with asphyxia-related complications at birth.

	No. of children	No. of cases	Rate/10,000 child -years	HR (95% CI)	Adjusted HR (95% CI)^b^
**Overall cardiovascular diseases**					
**Total**	2,826,424	4165	0.93		
Composite Asphyxia-related complications	31,419	90	1.80	1.96 (1.59–2.41)	1.90 (1.54–2.34)
Apgar score 0–3 at 1 min	26,238	54	1.34	1.53 (1.17–2.00)	1.48 (1.13–1.94)
Apgar score 0–3 at 5 min	4396	16	2.13	2.03 (1.24–3.32)	2.04 (1.25–3.34)
Neonatal Seizure^c^	5718	40	4.23	4.27 (3.13–5.83)	4.10 (3.00–5.60)
**Stroke**					
**Total**	2,826,424	1978	0.44		
Composite asphyxia at birth	31,419	51	1.02	2.34 (1.77–3.09)	2.29 (1.74–3.03)
Apgar score 0–3 at 1 min	26,238	26	0.64	1.53 (1.04–2.25)	1.48 (1.01–2.19)
Apgar score 0–3 at 5 min	4396	7	0.93	1.92 (0.92–4.04)	1.94 (0.92–4.07)
Neonatal Seizure^c^	5718	25	2.64	5.75 (3.88–8.53)	5.61 (3.78–8.33)
**Heart failure**					
**Total**	2,826,424	769	0.17		
Composite asphyxia at birth	31,419	19	0.38	2.25 (1.43–3.54)	2.17 (1.37–3.42)
Apgar score 0–3 at 1 min	26,238	12	0.30	1.86 (1.05–3.29)	1.79 (1.01–3.17)
Apgar score 0–3 at 5 min	4396	8	1.06	5.47 (2.73–10.98)	5.42 (2.70–10.87)
Neonatal Seizure^c^	5718	9	0.95	5.18 (2.69–9.99)	4.93 (2.55–9.53)
**Atrial fibrillation**					
**Total**	2,826,424	1186	0.26		
Composite asphyxia at birth	31,419	19	0.38	1.45 (0.92–2.28)	1.38 (0.87–2.17)
Apgar score 0–3 at 1 min	26,238	13	0.32	1.33 (0.77–2.29)	1.26 (0.73–2.18)
Apgar score 0–3 at 5 min	4396	<5^a^	0.40		
Neonatal Seizure^c^	5718	6	0.63	2.16 (0.97–4.82)	1.99 (0.89–4.44)
**Coronary heart disease**					
**Total**	2,826,424	376	0.08		
Composite asphyxia at birth	31,419	<5^a^	0.06		
Apgar score 0–3 at 1 min	26,238	<5^a^	0.07		
Apgar score 0–3 at 5 min	4396	0	0.00		
Neonatal Seizure^c^	5715	0	0.00		

Term singleton non-malformed live births in Sweden, 1988–2018.

a Exact count <5 is suppressed for confidentiality reasons.

b Adjusted for maternal age, country of origin, education level, cohabitation with a partner, parity, height, smoking during pregnancy, child's sex and year of delivery.

c Neonatal Seizure also included hypoxic ischemic encephalopathy.

**Fig. 1:**
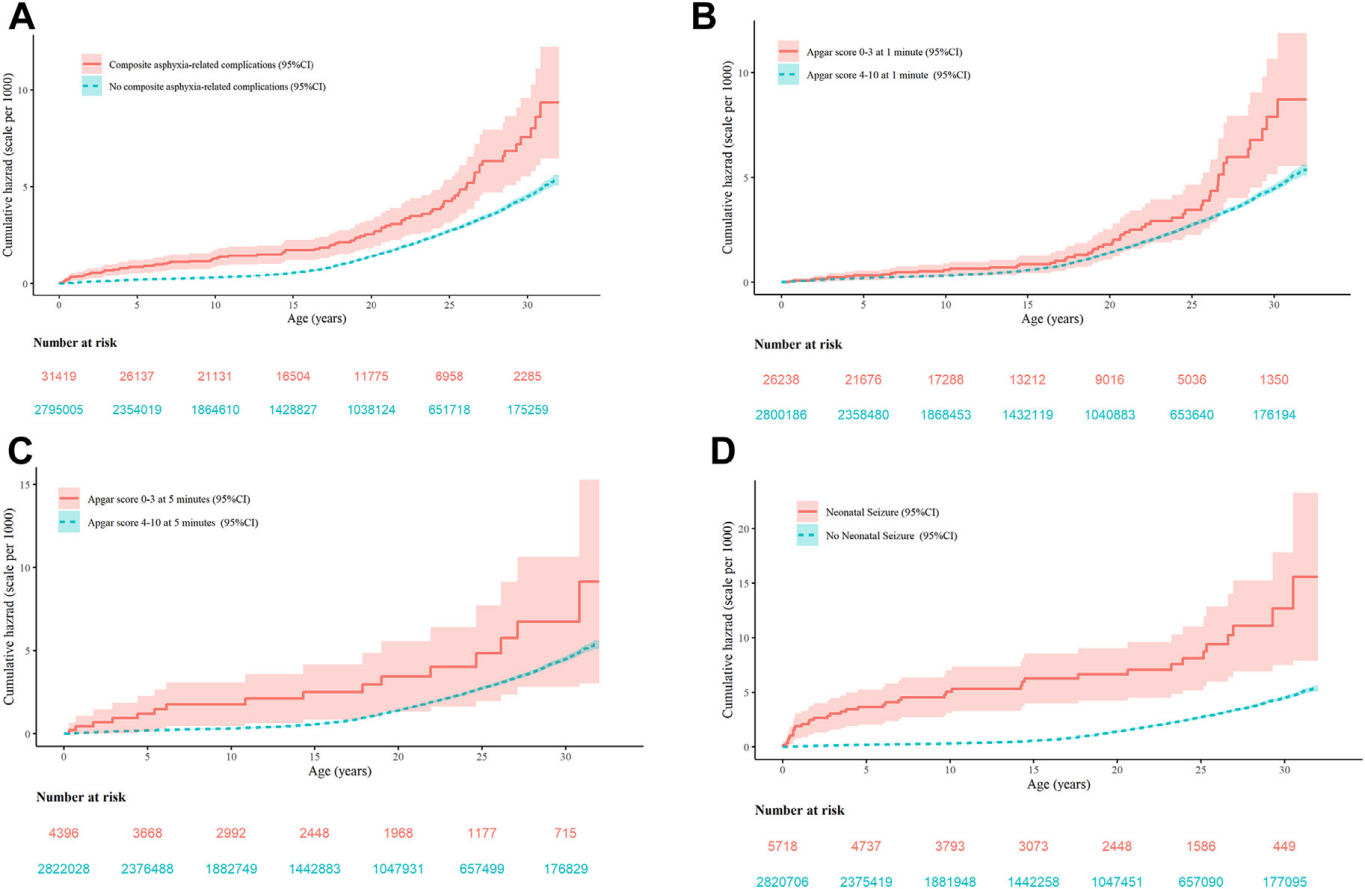
Unadjusted cumulative hazard curves show the cumulative hazard for cardiovascular disorders among individual exposed to a) asphyxia-related complications, b) Apgar score 0–3 at 1-min, c) Apgar score 0–3 at 5-min, and d) neonatal seizures (including hypoxic ischemic encephalopathy). Shading around the lines indicates the 95% CI.

The incidence rate of CVD increased with age, and unadjusted cumulative hazard curves showed a significantly higher cumulative hazard of cardiovascular disease among individuals with asphyxia-related complications (Fig. 1). The adjusted hazard ratios (HRs) of CVD were higher for individuals with Apgar score 0–3 at 1 min and at 5 min, and neonatal seizures (HRs were 1.48, 2.04, and 4.10, respectively) compared with those without such conditions (Table 2). Further adjustment for birth weight, and maternal BMI did not influence the results (Table S6). In the stratified analysis by child's sex (Table S7), child's sex did not modify the association asphyxia-related complication (P-value test for interaction 0.30). However, female infants with asphyxia-related complications had slightly higher rates of CVD compared with male with asphyxia-related complications.

The majority of CVD cases were due to stroke (mainly hemorrhagic stroke), followed by atrial fibrillation (Table 2). Asphyxia-related complications were associated with two-fold higher adjusted hazard ratios of stroke, and heart failure. Low Apgar score at 1 min, and especially low Apgar score at 5 min, were also associated with increased rates of stroke and heart failure (Table 2). Compared with individuals without neonatal seizures, having neonatal seizures was associated with a 5-fold increase in hazard ratio of stroke, and heart failure.

## Discussion

In this nationwide cohort study of more than 2.8 million individuals, we found that non-malformed term infants experiencing asphyxia-related complications at birth had higher risks of cardiovascular diseases in childhood and early adulthood. In particular, risks of stroke, and heart failure were increased, although absolute risks were low. These associations remained strong even after adjustment for birth weight and potential confounders. Especially those with Apgar score 0–3 at 5 min and those with neonatal seizures – indicating moderate to severe hypoxic-ischemic injury – had substantially increased risks of cardiovascular diseases.

Our finding of an association between asphyxia-related complications and increased risk of cardiovascular diseases later in life has not been demonstrated before.^28^^,^^29^ In animal models, neonatal hypoxia-ischemia has been shown to cause long-term cardiac dysfunction and ultrastructural deteriorations in adult rat myocardium, which provide some suggestions of an explanation of our findings.^30^

Low birth weight has previously been associated with increased risks of ischemic heart disease and stroke in later life.^31^ Besides genetic predisposition, fetal undernutrition has been suggested as an underlying causal mechanism for this association.^32^ However, adjustment for birth weight did not alter our results, indicating that asphyxia and hypoxic-ischemic injury to the heart and vascular tree may be independent risk factors for later CVD. Elucidating the etiology and identifying possible preventable factors for cardiovascular diseases in this group of high risk infants may therefore be of high clinical relevance.

Asphyxia typically triggers events within the cardiovascular system, ranging from heart rate and systemic blood pressure variations to more profound changes, like myocardial dysfunction, myocardial ischemia, and pulmonary artery hypertension.^33^ Asphyxiated neonates may have a decreased cardiac output state with ventricular myocardial dysfunction, decreased left ventricular preload, which is a consequence of pulmonary hypertension, and decreased ability to regulate vessel tone, potentially leading to long-term morbidity.^34^ Furthermore, hypoxic conditions delay the transition from anaerobic to aerobic energy production, which in turn makes the myocyte's contraction less efficient.^33^^,^^34^ The main physiological responses of the cardiovascular system to hypoxia is the redistribution of blood flow to vital organs, mainly heart and brain.^35^ However, this may be insufficient in cases of severe asphyxia, as both brain and myocardium develop ischemic lesions. As a result, this kind of cardiac insult may have a long-standing effects with limited reserve capacity when the circulatory system is challenged later in life.^36^ Furthermore, cardiovascular conditions related to acute systemic illnesses and arteriopathies could also be more common after asphyxia insult at birth. Given our findings, future studies with follow-up of cardiovascular structure and function at various ages are warranted to understand how and when cardiovascular complications manifest in children who were neonatally exposed to asphyxia.

Our data also provide some reassurance that the cardiovascular system may be more resilient to asphyxia insult at birth than the brain. For instance, among children born between 2015 and 2019 in Sweden, those who were treated with therapeutic hypothermia for asphyxia (242/401), after 2 years of follow-up, 16% needed habilitation services, 12% were diagnosed with cerebral palsy, and 7% suffered from developmental delay.^37^ On the other hand, in our study, after 30 years of follow-up, among 5718 cases of neonatal seizure and/or HIE, only 0.7% had a diagnosis for cardiovascular disease.

The key strengths of our study are the long and virtually complete follow-up, outcome data were collected prospectively and independent of exposure data, and the use of multiple health and population-based data sources. Information on exposures and outcomes and other high-quality registry data minimize the possibility of selection and information bias. We also adjusted for a number of important parental confounders, including maternal body mass index, and smoking during pregnancy. In addition, to avoid measuring CVD as a neonatal or infancy complication, all individuals diagnosed with CVD before 1 year of age were excluded. Lastly, we excluded all individuals with congenital malformations during the study period.

This study is also subject to some limitations. First, our cohort only allowed for a follow-up time reaching into young adulthood, and conclusions about possible associations between asphyxia complications and cardiovascular events should be restricted to this age period. Second, the validity of reported CVD diagnoses may be a concern. Nevertheless, the positive predictive values for stroke and acute myocardial infarction diagnoses are very high in the Swedish National Patient Register.^38^ Third, several of the outcomes had small numbers and the magnitude of the absolute risks were low. Fourth, reversed causation – i.e., that an existing cardiovascular, such as valvular heart disease, condition contributed to asphyxia-related complications – cannot be excluded. However, considering that congenital malformations and cases of CVD were excluded, this possibility is highly unlikely. Fifth, we did not have information on therapeutic hypothermia (introduced in 2007) for our entire cohort. Among those with HIE and CVD (n = 5), none had received therapeutic hypothermia. Therefore, we could not evaluate if therapeutic hypothermia modified the association between asphyxia-related complications and CVD.

Although the present findings provide support that asphyxia-related complications, especially low Apgar score and neonatal seizures, are associated with cardiovascular morbidity later in life, the absolute risk of CVD, at least in young age, is very low. Our findings need to be replicated, and possible underlying mechanisms, linking asphyxia-related complications to CVD, need to be identified.

## Contributors

Drs Razaz had full access to all of the data in the study and take full responsibility for the integrity of the data and the accuracy of the data analyses.

Study concept and design: Razaz, Norman, Cnattingius.

Acquisition of data: Razaz.

Drafting of the manuscript: Razaz.

Critical revision of the manuscript for important intellectual content: Razaz, Norman, Alfvén, Cnattingius.

Statistical analysis: Razaz.

Obtained funding: Razaz.

## Data sharing statement

The data sets generated and analyzed during the current study are not publicly available. The Swedish Secrecy Act does not permit data sharing of data sets based on individualized personal data.

## Declaration of interests

We declare no competing interests.
