# Supplementary material for: Low Apgar score and asphyxia complications at birth and risk of longer-term cardiovascular disease: a nationwide population-based study of term infants
Source: Lancet Reg Health Eur. 2022 Nov 3;24:100532. doi: 10.1016/j.lanepe.2022.100532 (PMC9832274; doi:10.1016/j.lanepe.2022.100532)
Supplement: Multimedia component 1 [file mmc1.docx]

**Supplementary Web Appendix**

**Low Apgar score and asphyxia complications at birth and risk of longer-term cardiovascular disease. A nationwide cohort study of term infants**

| **Table of Contents** | **Page** |
| --- | --- |
| **Table S1.** ICD-9 and ICD-10 codes for maternal and neonatal diseases. | **2** |
| **Table S2.** Frequency of cardiovascular diseases by ICD codes, before and after excluding cases with malformations. | **3** |
| **Table S3.** Matrix displaying overlap between the exposures | **4** |
| **Table S4.** Age at diagnoses of cardiovascular diseases | **5** |
| **Table S5.** Hazard Ratios of Cardiovascular disease According to Maternal Characteristics, Diseases, Pregnancy Complications, and Neonatal Characteristics, term singleton live births in Sweden, 1988-2018 | **6** |
| **Table S6.** Asphyxia-related complications and adjusted hazard ratios of cardiovascular disease. Term singleton non-malformed live births in Sweden, 1988-2018 | **8** |
| **Table S7.** Asphyxia-related complications and rates of cardiovascular disease stratified by child’s sex. Term singleton non-malformed live births in Sweden, 1988-2018. | **9** |

**Table S1.** ICD-9 and ICD-10 codes for maternal and neonatal conditions.

| **Diseases** | **ICD-9 codes** | **ICD-10 codes** |
| --- | --- | --- |
| **Maternal complications** |  |  |
| Pre-gestational hypertension | 401-405, 642A-C, 642H +checkbox* | I10-I15, O10 and O11 +checkbox* |
| Preeclampsia and eclampsia | 642E-642G | O14 and O15 |
| Pregestational diabetes | 648W | E10-E14, O24.0-O24.3 |
| Gestational diabetes | 250, 648A | O24.4 |
| **Malformation^†^** | 740-759 | Q00-Q99 |
| **Asphyxia at birth ^‡^** |  |  |
| Neonatal Seizure | 779A | P90 |
| Hypoxic ischemic encephalopathy | 779B, 779C | P913-P916 |

Diseases were defined using the Swedish versions of the International Classification of Diseases, ninth and tenth revisions (ICD-9; ICD-10). Sweden used the ninth revision (ICD-9) from 1987 through 1996, and the tenth revision (ICD-10) has been used since 1997.

^*^Pre-gestational hypertension is also recorded in a checkbox in the prenatal record at first prenatal visit.

^†^Diagnosis of malformations is derived from the Medical Birth Register or the Patient Register (also including out-patient hospital care from 2001) at any age during the follow-up.

^‡^Diagnoses of asphyxia conditions are from Medical Birth Register or the Patient Register (in-patient hospital care, admission date at 0-27 days of age).

| **Table S2.** Frequency of cardiovascular diseases by ICD codes, before and after excluding cases with malformations. | | | | |
| --- | --- | --- | --- | --- |
| **Cardiovascular diseases** | **ICD 9/10 Codes** | **Overall cases** | **Cases also with congenital malformations^a^** | **Cases with no congenital malformations** |
|  |  | N | N (%) | N |
| Stroke | 430-436, I60-I65 | 2591 | 667 (26%) | 1978 |
| Coronary heart disease | 410-414, I20-I25 | 439 | 132 (30%) | 376 |
| Acute myocardial infraction | 410, I20 | 132 | 32 (24%) | 100 |
| Heart failure | 428, I50 | 1081 | 363 (34%) | 769 |
| Atrial fibrillation | 427D, I48 | 1472 | 253 (17%) | 1186 |

**^a^**Both minor and major malformation were included, ICD 9: 740-759; ICD 10: Q00-Q99

| **Table S3.** Matrix displaying overlap between the exposures | | | | | |
| --- | --- | --- | --- | --- | --- |
| **Neonatal Seizure** | **hypoxic ischemic encephalopathy** | **Apgar score 0-3 at 1 minute** | **Apgar score 0-3 at 5 minutes** | **Number** | **Percent (%)** |
| **0** | **0** | **1** | **0** | 22135 | 70.45 |
| **1** | **0** | **0** | **0** | 2527 | 8.04 |
| **0** | **0** | **1** | **1** | 2101 | 6.69 |
| **0** | **0** | **0** | **1** | 1465 | 4.66 |
| **0** | **1** | **0** | **0** | 976 | 3.11 |
| **1** | **0** | **1** | **0** | 616 | 1.96 |
| **0** | **1** | **1** | **0** | 435 | 1.38 |
| **1** | **0** | **1** | **1** | 300 | 0.95 |
| **0** | **1** | **1** | **1** | 267 | 0.85 |
| **1** | **1** | **1** | **1** | 202 | 0.64 |
| **1** | **1** | **1** | **0** | 182 | 0.58 |
| **1** | **1** | **0** | **0** | 152 | 0.48 |
| **1** | **0** | **0** | **1** | 42 | 0.13 |
| **0** | **1** | **0** | **1** | 11 | 0.04 |
| **1** | **1** | **0** | **1** | 8 | 0.03 |

| **Table S4.** Age at diagnoses of cardiovascular diseases | | | | | | | | | | | | |
| --- | --- | --- | --- | --- | --- | --- | --- | --- | --- | --- | --- | --- |
| **Age of diagnosis (years)** | **All Cardiovascular diseases** | | **Stroke** | | **Heart Failure** | | **Coronary heart disease** | | **Acute myocardial infraction** | | **Atrial fibrillation** | |
|  | **No.** | **%** | **No.** | **%** | **No.** | **%** | **No.** | **%** | **No.** | **%** | **No.** | **%** |
| 1-4 | 471 | 11.31 | 297 | 15.02 | 107 | 13.91 | 70 | 18.62 | 11 | 14.17 | 11 | 0.93 |
| 5-8 | 223 | 5.59 | 180 | 9.1 | 29 | 3.77 | 15 | 3.99 | 2 | 2.36 | 12 | 1.01 |
| 9-12 | 211 | 5.07 | 144 | 7.28 | 31 | 4.03 | 19 | 5.05 | 1 | 1.57 | 21 | 1.77 |
| 13-16 | 425 | 10.2 | 265 | 13.4 | 62 | 8.06 | 35 | 9.31 | 3 | 7.87 | 77 | 6.49 |
| 17-20 | 888 | 21.32 | 358 | 18.1 | 152 | 19.77 | 82 | 21.81 | 16 | 16.54 | 335 | 28.25 |
| ≥21 | 1937 | 46.51 | 734 | 37.11 | 388 | 50.46 | 155 | 41.22 | 66 | 57.48 | 730 | 61.55 |

| **Table S5. Hazard Ratios of Cardiovascular disease According to Maternal Characteristics, Diseases, Pregnancy Complications, and Neonatal Characteristics, term singleton live births in Sweden, 1988-2018** | | |
| --- | --- | --- |
| **Characteristics** | **Hazard ratios (95% CI)** | |
|  | **Unadjusted** | **Adjusted** |
| **Total** |  |  |
| **Maternal age (years)** |  |  |
| ≤19 | 1.12 (0.93-1.36) | 1.04 (0.85-1.26) |
| 20-24 | Ref. | Ref. |
| 25-29 | 0.87 (0.81-0.95) | 0.90 (0.83-0.98) |
| 30-34 | 0.87 (0.79-0.94) | 0.89 (0.81-0.98) |
| ≥35 | 0.84 (0.75-0.93) | 0.85 (0.75-0.95) |
| **Country of birth** |  |  |
| Sweden | Ref. | Ref. |
| Other Nordic | 1.08 (0.91-1.28) | 1.07 (0.90-1.27) |
| Non-Nordic | 1.00 (0.90-1.11) | 0.99 (0.89-1.10) |
| Data missing |  |  |
| **Education (years)** |  |  |
| ≤9 | 1.40 (1.25-1.56) | 1.32 (1.17-1.48) |
| 10-11 | 1.20 (1.10-1.30) | 1.16 (1.06-1.26) |
| 12 | 1.17 (1.07-1.29) | 1.14 (1.03-1.25) |
| 13-14 | 1.13 (1.02-1.24) | 1.13 (1.02-1.25) |
| ≥15 | Ref. | Ref. |
| Data missing |  |  |
| **Mother cohabits with partner** |  |  |
| Yes | Ref. | Ref. |
| No | 1.29 (1.14-1.47) | 1.17 (1.03-1.33) |
| Data missing |  |  |
| **Parity** |  |  |
| 1 | Ref. | Ref. |
| 2 | 0.95 (0.88-1.01) | 0.98 (0.91-1.05) |
| 3 | 0.97 (0.89-1.07) | 1.02 (0.92-1.12) |
| ≥4 | 1.00 (0.88-1.13) | 1.02 (0.89-1.17) |
| **Maternal height (cm)** |  |  |
| ≤159 | 0.97 (0.88-1.08) | 0.95 (0.85-1.06) |
| 160-164 | 0.93 (0.85-1.01) | 0.92 (0.84-1.00) |
| 165-169 | Ref. | Ref. |
| ≥170 | 1.06 (0.98-1.14) | 1.07 (0.99-1.16) |
| Data missing |  |  |
| **Maternal BMI** |  |  |
| <18.5 | 1.06 (0.87-1.29) | 1.01 (0.83-1.23) |
| 18.5-24.9 | Ref. | Ref. |
| 25-29.9 | 1.12 (1.02-1.23) | 1.11 (1.01-1.22) |
| 30-34.9 | 1.18 (1.01-1.39) | 1.15 (0.98-1.35) |
| ≥35 | 1.61 (1.27-2.04) | 1.55 (1.22-1.96) |
| Missing |  |  |
| **Smoking** |  |  |
| No | Ref. | Ref. |
| Yes | 1.27 (1.18-1.37) | 1.20 (1.11-1.29) |
| Data missing |  |  |
| **Diabetic disease** |  |  |
| No | Ref. | Ref. |
| Gestational diabetes | 1.06 (0.76-1.48) | 1.07 (0.76-1.49) |
| Pregestational diabetes | 1.06 (0.63-1.78) | 1.06 (0.62-1.78) |
| **Hypertensive disease** |  |  |
| No | Ref. | Ref. |
| Pregestational hypertension | 0.90 (0.57-1.41) | 0.91 (0.58-1.43) |
| Preeclampsia | 1.14 (0.93-1.39) | 1.14 (0.93-1.39) |
| **Maternal cardiovascular disorder** |  |  |
| No | Ref. | Ref. |
| Yes | 0.84 (0.32-2.25) | 0.82 (0.31-2.19) |
| **Paternal cardiovascular disorder** |  |  |
| No | Ref. | Ref. |
| Yes | 1.36 (0.82-2.25) | 1.34 (0.81-2.23) |
| **Year of delivery** |  |  |
| 1988-1994 | 1.09 (0.80-1.49) | 1.01 (0.74-1.37) |
| 1995-1999 | 0.96 (0.70-1.32) | 0.92 (0.67-1.26) |
| 2000-2004 | 1.15 (0.83-1.58) | 1.13 (0.82-1.55) |
| 2005-2009 | 1.27 (0.91-1.76) | 1.27 (0.92-1.76) |
| 2010-2014 | 1.32 (0.94-1.84) | 1.32 (0.94-1.84) |
| 2015-2018 | Ref. | Ref. |
| **Mode of delivery** |  |  |
| Vaginal non-instrumental | Ref. | Ref. |
| Vaginal instrumental | 1.23 (1.07-1.41) | 1.20 (1.05-1.38) |
| Elective cesarean section | 1.21 (1.04-1.41) | 1.23 (1.05-1.43) |
| Emergency cesarean section | 1.21 (1.04-1.41) | 1.20 (1.03-1.40) |
| Data missing |  |  |
| **Newborn’s sex** |  |  |
| Male | 1.70 (1.60-1.81) | 1.70 (1.60-1.81) |
| Female | Ref. | Ref. |
| **Birth weight for gestational age** |  |  |
| **(percentiles)** |  |  |
| <3 | 1.42 (1.15-1.76) | 1.37 (1.11-1.70) |
| 3 to <10 | 1.19 (1.04-1.36) | 1.17 (1.02-1.33) |
| 10 to 90 | Ref. | Ref. |
| >90 to 97 | 1.06 (0.95-1.18) | 1.07 (0.96-1.19) |
| >97 | 1.19 (1.02-1.40) | 1.20 (1.02-1.41) |
| Data missing |  |  |
| ^‡^ From a Cox proportional hazards model adjusted for maternal age, country of origin, education level, cohabitation with a partner, parity, height, smoking during pregnancy, child's sex, year of delivery. | | |

| **Table S6.** Asphyxia-related complications and adjusted hazard ratios of cardiovascular disease. Term singleton non-malformed live births in Sweden, 1988-2018 | | | |
| --- | --- | --- | --- |
| **Asphyxia conditions** | **Model 1** | **Model 2** | **Model 3** |
| Composite asphyxia at birth | 1.90 (1.54-2.34) | 1.86 (1.50-2.30) | 1.88 (1.53-2.32) |
| Apgar score 0-3 at 1 minute | 1.48 (1.13-1.94) | 1.44 (1.09-1.89) | 1.46 (1.12-1.91) |
| Apgar score 0-3 at 5 minutes | 2.04 (1.25-3.34) | 1.81 (1.07-3.07) | 2.03 (1.24-3.32) |
| Neonatal Seizures | 4.10 (3.00-5.60) | 6.04 (4.35-8.39) | 5.86 (4.22-8.14) |
| Model 1: From a Cox proportional hazards model adjusted for maternal age, country of origin, education level, cohabitation with a partner, parity, height, smoking during pregnancy, child's sex and year of delivery | | | |
| Model 2: In addition to the factors noted in model 1, also adjusted for birth weight | | |  |
| Model 3: In addition to the factors noted in model 1, also adjusted for maternal body mass index. | | | |

| **Table S7.** Asphyxia-related complications and rates of cardiovascular disease stratified by child’s sex. Term singleton non-malformed live births in Sweden, 1988-2018. | | | | | |
| --- | --- | --- | --- | --- | --- |
|  | **No. of children** | **No. of cases** | **Rate/10,000 child -years** | **Unadjusted hazard ratio (95% CI)** | **Adjusted hazard ratio (95% CI)** |
| **Female** | | | | | |
| **Total** | 1388962 | 1493 | 0.7 |  |  |
| Composite Asphyxia-related complications | 13081 | 31 | 1.5 | 2.21 (1.55-3.15) | 2.24 (1.57-3.20) |
| **Male** | | | | | |
| **Total** | 1437462 | 2672 | 1.2 |  |  |
| Composite Asphyxia-related complications | 18338 | 59 | 2.0 | 1.75 (1.35-2.27) | 1.77 (1.36-2.29) |
| Adjusted for maternal age, country of origin, education level, cohabitation with a partner, parity, height, smoking during pregnancy, child's sex and year of delivery. | | | | | |
